# Total fat and omega-3 fatty acids intake in relation to serum brain-derived neurotrophic factor (BDNF) levels and psychological disorders in Iranian adults

**DOI:** 10.1038/s41598-023-32510-x

**Published:** 2023-04-03

**Authors:** Sedigheh Bahadorpour, Zahra Hajhashemy, Sobhan Mohammadi, Elahe Mokhtari, Zahra Heidari, Parvane Saneei

**Affiliations:** 1 Department of Community Nutrition, School of Nutrition and Food Science, Nutrition and Food Security Research Center, Isfahan University of Medical Sciences, P.O. Box 81745-151, Isfahan, Iran; 2 Student Research Committee, Isfahan University of Medical Sciences, Isfahan, Iran; 3 Department of Biostatistics and Epidemiology, School of Health, Isfahan University of Medical Sciences, Isfahan, Iran

**Keywords:** Neurological disorders, Nutrition disorders

## Abstract

Considering contradictory findings of previous investigations and growing prevalence of psychological disorders, we investigated association between dietary total fat and omega-3 fatty acids intake with serum brain-derived neurotrophic factor (BDNF) levels, depression, anxiety and psychological distress in Iranian adults. Using a multistage cluster random sampling method, 533 middle-aged adults were included in this cross-sectional study. A validated semi-quantitative 168-item food frequency questionnaire was used to examine dietary intakes. A 12-h fasting blood sample was drawn to measure serum BDNF. Serum BDNF values in the first decile were considered low level. Hospital Anxiety and Depression Scale (HADS) and General Health Questionnaire (GHQ) were used to assess depression, anxiety and psychological distress. A U-shaped relationship between fat intake and prevalence of anxiety and distress was found. The third quartile of fat intake compared to the first quartile was significantly related to 80% decreased odds of depression (OR = 0.20, 95% CI 0.05–0.80), in fully-adjusted model. Participants in the third quartile of fat intake compared to those in the first quartile had significantly 45% lower odds for distress, in the crude model (OR = 0.55, 95% CI 0.33–0.92); however, this association disappeared after considering confounders. There was no significant association between omega-3 fatty acids intake and odds of depression, anxiety or distress. Prevalence of low-BDNF values was higher in participants with depression, as compared to non-depressed subjects (14.9 vs. 9%; *P* = 0.06). This cross-sectional study illustrated a U-shaped relationship between fat intake and prevalence of anxiety and distress. Moderate intake of fat was related to lower odds of depression. Prevalence of low-BDNF values was slightly higher in subjects with depression compared to non-depressed individuals.

## Introduction

Depression, anxiety, and psychological distress are the most prevalent mental disorders that many people suffer from worldwide^1^. Depression and anxiety have respectively influenced approximately 4.7 and 7.3% of the population around the world^1,2^; women are more prone to psychological disorders than men^3^. Prevalence of depression in Iran was approximately 30%^4^. These psychiatric disorders have adverse effects on quality of life and are related to obesity, inflammation, cardiovascular disease (CVD), myocardial infarction (MI), and suicidal behaviors^5–8^. Thus, these disorders can eventually lead to an increased mortality rate^9^.

Susceptibility to depression is influenced by sex, genetic, environmental and endocrine factors^10,11^. Diet is one of the modifiable environmental parameters that can play a role in treatment and management of psychological disorders symptoms^12–14^. Since central nervous system (CNS) has the highest concentration of fatty acids after adipose tissue, mood status is drastically influenced by cell membrane fat content^15,16^. Previous studies have shown that dietary intake of omega-3 fatty acids was associated with mental disorders such as anxiety and depression^17–19^. Brain-derived neurotrophic factor (BDNF), one of the key regulators of growth and function of nervous system, is a member of the family of secretory proteins including nerve growth factor (NGF), neurotrophin 3 and 4. BDNF plays a role in differentiation, growth of neurons, synapse formation and cognitive functions^20^. Some pieces of evidence suggested that serum BDNF levels are reduced in patients with depression and anxiety^21,22^. Some antidepressant drugs can improve mood by increasing BDNF gene expression^23^.

Some prior investigations have suggested that omega-3 fatty acids intake may have a beneficial effect on depression and anxiety disorders^24,25^, but others did not confirm this impact or reported different findings in men and women^26,27^. On the other hand, the relationship between usual dietary fat and omega-3 fatty acids intake with serum BDNF levels has been understudied yet; most previous investigations in this regard have examined the effect of omega-3 fatty acids supplementation on BDNF levels, using a randomized clinical trial (RCT) design^28,29^. Considering contradictory findings of previous investigations and growing prevalence of psychological disorders, we decided to investigate association between dietary total fat and omega-3 fatty acids intake with serum BDNF levels, depression, anxiety and psychological distress in Iranian adults.

## Methods and materials

### Study design and participants

This population-based cross-sectional study was conducted in 2021 and included a representative sample of Iranian adults living in a large central city of Iran (Isfahan). By taking a confidence interval of 95%, precision (d) of 4% and 30% prevalence of depression among Iranian adults^4^ into account, a least sample size of 505 was acceptable for this investigation. Since covid-19 pandemic was very prevalent during our data collection, we invited a total of 600 individuals to participate in the study. Using a multistage cluster random sampling method, 20 schools in different educational districts of Isfahan city were selected. In order to have a representative sample of general adults with different socioeconomic statuses, all individuals who were working in these selected schools (including school managers, employees, assistants, teachers and crews) were invited to participate in the study. Nevertheless, we did not include persons who, (1) were following a special diet; (2) were pregnant or in a lactating period; and (3) had a prior history of type 1 diabetes, stroke, cardiovascular disease, and cancer. Among 600 invited individuals, 543 agreed to participate. Ten other participants were excluded because (1) more than 70 items of their FFQ were blank (n = 4); (2) they reported total energy intake of more than 4200 or less than 800 kcal/d (n = 3); (3) they did not complete questionnaires of psychological disorders (n = 3). Therefore, the current analysis was conducted on 533 individuals. Since patients with aggressive psychological disorders are usually unable or not allowed to work, only 5.2% of the study participants (n = 28) were taking anti-psychotic medications. Therefore, these few patients with controlled status were included in the analyses and then taking anti-psychotic drugs was considered as a confounder in the analyses. All participants signed written informed consent. The protocol of the study was ethically approved by the local Ethics Committee of Isfahan University of Medical Sciences (no. IR.MUI.RESEARCH.REC.1399.612). The study procedure was performed according to the declaration of Helsinki and STROBE checklist.

### Assessment of dietary intakes

Dietary intakes were assessed using a semi-quantitative 168-item food frequency questionnaire (FFQ), which was designed and validated specifically for Iranian adults^30^. A previous validation study of this FFQ examined dietary intakes of 132 middle-aged adults using this FFQ in comparison with multiple 24-h dietary recalls. The correlation coefficients between dietary intakes obtained from twelve 24-h dietary recalls and this FFQ were 0.59 for fat, 0.55 for total energy intake, 0.65 for proteins, 0.65 for magnesium and 0.67 for fiber. The reliability of this FFQ was also assessed by comparing nutrient intakes obtained from the FFQ on two occasions 1-year apart. Overall, the mentioned validation study revealed that this FFQ could be a reasonably valid and reliable tool for assessment of usual dietary intakes among Iranian adults^30^. Participants were instructed to complete this FFQ by an expert dietitian. They were asked to report the frequency and amount of their food intake in the preceding year. Then, we converted portion sizes of consumed foods to gram per day by the use of household measures. Finally, we obtained daily intake of energy and all nutrients by entering all food items into Nutritionist IV software. Omega-3 fatty acids intake was estimated by summing the intakes of eicosapentaenoic acid (EPA), docosahexaenoic acid (DHA) and alpha-linolenic acid (ALA).

### Assessment of psychological disorders and serum BDNF values

A validated Persian version of Hospital Anxiety and Depression Scale (HADS) was used to assess anxiety and depression^31^. This brief and useful questionnaire includes 7 items for anxiety subscale and 7 items for depression subscale. Each item consists of a four-point option with a score of 0–1–2–3. Therefore, a score in the range of 0–21 is obtained for anxiety and depression. Higher scores indicate higher levels of anxiety and depression. A score of 7 or less was defined as normal status, while a score of 8 or more was considered as having anxiety or depression. A 12-item validated Persian version of General Health Questionnaire (GHQ) questionnaire was used to assess psychological distress levels^32^. This simple short-form questionnaire requests individuals to report their recent experience of psychological distress. Each item has 4 options (less than usual, normal, more than usual and much more than usual). There are two methods for scoring this questionnaire, the bimodal (0–0–1–1) and the Likert method (0–1–2–3), which provide a total score of 12 or 36, respectively. In the present study, the bimodal scoring method was applied. A higher score was defined as a greater degree of psychological distress. In the current study, the score in the range of 0 to 3 was considered low psychological distress, while a score of 4 or more was considered high psychological distress.

The blood samples were derived after a 12-h overnight fasting status. A separated serum was provided by centrifuging the clot sample. Serum BDNF values were measured using commercial ELISA kits (Zellbio, Veltlinerweg, Germany). The assay range for BDNF was from 0.312 to 20 ng/mL with a sensitivity of 0.078 ng/mL. Intra-assay and inter-assay CV were respectively < 8% and < 10%. Based on a previous study^33^, we ranked participants based on deciles of serum BDNF concentrations and considered those in the first decile of BDNF values (serum BDNF level < 0.47 ng/mL) as individuals with low serum BDNF values.

### Assessment of other variables

Weight was measured with light clothing, without shoes by the use of a body composition analyzer (Tanita MC-780MA, Tokyo, Japan), with an accuracy of 0.1 kg. Height was measured without shoes using a tape measure mounted on the wall. Body mass index (BMI) was calculated by dividing weight in kilogram by height squared in meter (kg/m^2^). Using a digital sphygmomanometer (OMRON, M3, HEM-7154-E, Japan) with an accuracy of 0.5 mmHg, blood pressure was measured twice for each participant after a 5-min of resting time in a sitting position; the mean of these measurements was recorded.

Physical activity was assessed using the validated International Physical Activity Questionnaire (IPAQ)^34^. A self-administered questionnaire was used to examine the socio-economic status (SES) including job, number of family members, head of household, job of the household head, home ownership, type of house, number of family cars, type of cars, number of laptops, number of travels in the year (within the country or abroad), approximate income of the participant and approximate income of the household. Scores of these items were summed to determine a total score of SES. Then, individuals were ranked into three levels low, medium and high SES status. Information of other variables such as age, sex, smoking, medical history, marital status and education were collected using a self-administered questionnaire. Data of anti-depressant drugs intake (including nortriptyline, amitriptyline, imipramine, fluoxetine, citalopram and fluvoxamine) and dietary supplements intake (including vitamin D, omega-3, B complex, vitamin B1, B2, B3, B6, multivitamin, neurobion, calcium and iron) that were related to mental disorders were also gathered.

### Statistical methods

Normality distribution of variables was examined using Kolmogorov–Smirnov test. Data were reported as mean ± SD/SE for continuous variables and percentage for categorical variables. First, subjects were ranked into quartiles of fat intake (as percentage of total energy intake) and energy-adjusted omega-3 fatty acids intake. Chi-square test and one-way analysis of variance (ANOVA) were used to compare the categorical and continuous variables across quartiles of fat and omega-3 fatty acids intake. Using analysis of covariance (ANCOVA), we reported the sex, age, and energy-adjusted dietary intakes of subjects across quartiles of fat and omega-3 fatty acids intake. Binary logistic regression was used to report odds ratio (OR) and 95% confidence interval (95% CI) for psychological disorders across quartiles of exposures, in crude and multivariable-adjusted models. In the first model, adjustments were done for energy intake, age and sex. The effects of marital status, education, physical activity, smoking, hypertension, diabetes, anti-depressant drugs intake, dietary supplements intake and SES were additionally controlled in the second model. Further adjustment for BMI was done in the last model. The first quartile of fat or omega-3 fatty acids intake was considered the reference category in all models. Quartiles of fat or omega-3 fatty acids intake were treated as continuous variables to determine the trends in logistic regression models. Moreover, we reported crude and adjusted (for age, sex and physical activity) mean values of serum BDNF concentrations (± SE) in quartiles of fat and omega-3 fatty acids intake, by the use of ANOVA and ANCOVA. Chi-square fisher's exact test was used to report prevalence of low-BDNF values (decile 1 of BDNF or values < 0.47 ng/mL) in participants with and without psychological disorders. All statistical analyses were performed using the SPSS software version 21 (SPSS Inc., version 21.0, Chicago, IL). *P* values less than 0.05 were considered statistically significant.

### Ethical approval and consent to participate

The study procedure was performed according to declaration of Helsinki and STROBE checklist. All participants provided an informed written consent. The study protocol was approved by the local Ethics Committee of Isfahan University of Medical Sciences (no. IR.MUI.RESEARCH.REC.1399.612).

## Results

A total of 533 Iranian adults were included in the current population-based cross-sectional study; 53.8% of them were men. The mean age, weight and BMI of participants were respectively 42.5 (± 11.15) yr, 75.7 (± 14.5) kg and 26.9 (± 4.41) kg/m^2^. Among them, 18.9, 5.1 and 33.4% have respectively suffered from depression, anxiety and high psychological distress. The average serum BDNF concentration in study participants was 1.25 ng/mL. The mean fat and omega-3 fatty acids intake were respectively 26.91 (± 0.30) (percentage of energy intake) and 0.14 (± 0.004) (g/d).

General characteristics and dietary intakes of study participants across quartiles of fat and omega-3 fatty acids intake are provided in Tables 1 and 2. Those in the top category of fat and omega-3 fatty acids intake were more likely to be women, younger, and had higher socioeconomic status and dietary supplement intake, compared to the bottom category. Subjects in the last quartile of fat intake had higher intakes of energy, protein, cholesterol, saturated fatty acid (SFA), monounsaturated fatty acid (MUFA), polyunsaturated fatty acid (PUFA), dairy, red and processed meat, and vitamin B6, and lower intakes of carbohydrates, total fiber, thiamin, iron, whole and refined and fruits, compared to those in the first quartile of fat intake. In case of omega-3 fatty acids intake, individuals in the top quartile of intake had higher intakes of cholesterol, SFA, MUFA, PUFA, vitamin B6, whole grains and vegetables, and lower intakes of carbohydrates, thiamin, iron and refined grains, as compared to the first category.

**Table 1:** General characteristics of study participants across quartiles of fat (as percentage of energy intake) and energy-adjusted omega-3 fatty acids intake^a^.

	Quartiles of fat intake (% of energy)					Energy-adjusted quartiles of omega-3 fatty acids intake (g/d)				
	Q1 (n = 132) (< 21.8%)	Q2 (n = 134) (21.8–26.7%)	Q3 (n = 133) (26.7–31.4%)	Q4 (n = 134) (> 31.4%)	*P*^b^	Q1 (n = 134) (< 0.08 g)	Q2 (n = 134) (0.08–0.13 g)	Q3 (n = 132) (0.13–0.19 g)	Q4 (n = 133) (> 0.19 g)	*P*^b^
Mean fat or omega-3 fatty acids intake	18.12 ± 2.9	24.39 ± 1.4	28.90 ± 1.2	36.12 ± 4.0		0.06 ± 0.01	0.10 ± 0.01	0.15 ± 0.01	0.27 ± 0.10	
Sex, (Male) (%)	74.2	55.2	47.4	38.8	< 0.001	69.4	51.5	53.0	41.4	< 0.001
Age (year)	45.90 ± 12.09	42.23 ± 10.51	42.17 ± 11.91	40.11 ± 9.20	< 0.001	45.02 ± 10.95	43.67 ± 10.54	40.78 ± 11.64	40.84 ± 10.97	0.01
Weight (kg)	75.96 ± 15.31	77.87 ± 15.04	75.10 ± 13.14	73.88 ± 14.28	0.14	75.10 ± 13.03	76.37 ± 16.53	76.33 ± 13.66	75.01 ± 14.64	0.78
BMI^c^ (kg/m^2^)	26.65 ± 4.79	27.50 ± 4.46	26.93 ± 4.37	26.52 ± 3.94	0.27	26.40 ± 3.88	27.38 ± 5.19	27.14 ± 3.84	26.67 ± 4.55	0.25
Physical activity (MET.min/wk)	1105.84 ± 186.16	1247.05 ± 231.12	896.21 ± 106.27	868.17 ± 102.26	0.31	875.88 ± 113.27	1259.11 ± 269.92	1046.70 ± 117.27	935.28 ± 99.00	0.37
Education (University graduated) (%)	84.1	90.3	91.0	87.3	0.28	85.8	85.8	89.4	91.7	0.35
Marital status (Married) (%)	80.8	80.3	83.3	85.1	0.15	83.5	80.5	80.9	84.7	0.86
Smoking status (Smokers) (%)	4.2	1.7	3.3	3.5	0.28	4.1	1.7	3.3	3.4	0.52
High SES status^d^ (%)	23.0	33.7	39.8	46.1	0.04	25.7	29.2	49.5	37.2	0.01
Anti-depressants medications use^e^ (%)	6.9	5.4	3.9	5.3	0.75	3.8	8.5	4.7	4.6	0.32
Dietary supplements use^f^ (%)	66.1	71.6	86.0	74.8	0.01	71.2	73.8	73.2	80.3	0.35
Diabetes (%)	6.9	6.7	3.8	3.0	0.35	6.0	3.0	5.3	6.0	0.64
Hypertension (%)	28.8	24.1	26.4	19.1	0.30	29.3	22.9	21.5	24.4	0.48
Overweight/obese (%)	65.2	73.1	68.4	64.9	0.44	67.2	67.9	71.2	65.4	0.78

^a^Continuous variables are reported as mean ± SD, except for physical activity that is reported as mean ± SE. Categorical variables are reported as percentage.

^b^*P*-values obtained from ANOVA and χ2 test for continuous and categorical variables, respectively.

^c^BMI: Body Mass Index.

^d^Socioeconomic status (SES) status was evaluated based on job, number of family members (more than 4 vs. 4 or less), head of household, the job of the household head, home ownership status, type of house, number of family cars, type of car, number of laptops, number of travels (within the country or abroad), the approximate income of the person and the approximate income of the household per month.

^e^Including nortriptyline, amitriptyline, imipramine, fluoxetine, citalopram and fluvoxamine.

^f^Including vitamin D, omega-3, B complex, vitamin B1, B2, B3, B6, multivitamin, neurobion, calcium and iron.

**Table 2:** Dietary intakes of study participants across quartiles of fat (as percentage of energy intake) and energy-adjusted omega-3 fatty acids intake^a^.

	Quartiles of fat intake (% of energy)					Energy-adjusted quartiles of omega-3 fatty acids intake (g/d)				
	Q1 (n = 132) (< 21.8%)	Q2 (n = 134) (21.8–26.7%)	Q3 (n = 133) (26.7–31.4%)	Q4 (n = 134) (> 31.4%)	*P*^b^	Q1 (n = 134) (< 0.08 g)	Q2 (n = 134) (0.08–0.13 g)	Q3 (n = 132) (0.13–0.19 g)	Q4 (n = 133) (> 0.19 g)	*P*^b^
Mean fat or omega-3 fatty acids intake	18.12 ± 2.9	24.39 ± 1.4	28.90 ± 1.2	36.12 ± 4.0		0.06 ± 0.01	0.10 ± 0.01	0.15 ± 0.01	0.27 ± 0.10	
Nutrient										
Energy, kcal	2142.47 ± 60.27	2292.33 ± 58.07	2244.68 ± 58.41	2416.40 ± 59.05	0.01	2178.90 ± 59.39	2276.58 ± 58.50	2350.02 ± 59.04	2293.84 ± 59.22	0.23
Protein, % of energy	12.84 ± 0.24	13.92 ± 0.23	15.36 ± 0.23	14.81 ± 0.23	< 0.001	14.12 ± 0.24	13.82 ± 0.24	14.48 ± 0.24	14.52 ± 0.24	0.15
Carbohydrate, % of energy	70.87 ± 0.37	63.71 ± 0.36	57.73 ± 0.36	51.01 ± 0.36	< 0.001	63.88 ± 0.69	61.46 ± 0.68	59.99 ± 0.69	57.83 ± 0.69	< 0.001
Cholesterol, mg	200.20 ± 9.59	256.15 ± 9.20	315.77 ± 9.25	331.94 ± 9.40	< 0.001	245.85 ± 10.17	264.74 ± 9.99	292.92 ± 10.10	301.83 ± 10.11	< 0.001
SFA, gr	14.82 ± 0.49	19.85 ± 0.47	23.59 ± 0.47	31.01 ± 0.48	< 0.001	20.51 ± 0.68	21.57 ± 0.67	23.22 ± 0.67	24.11 ± 0.67	0.01
MUFA, gr	14.66 ± 0.37	19.24 ± 0.35	23.25 ± 0.36	30.01 ± 0.36	< 0.001	18.55 ± 0.56	21.47 ± 0.55	22.31 ± 0.56	24.95 ± 0.56	< 0.001
PUFA, gr	11.01 ± 0.56	14.19 ± 0.53	17.20 ± 0.54	22.11 ± 0.55	< 0.001	14.29 ± 0.63	16.63 ± 0.62	15.73 ± 0.63	17.93 ± 0.63	0.01
Total fiber, gr	23.31 ± 0.55	22.00 ± 0.53	21.54 ± 0.53	17.81 ± 0.54	< 0.001	21.46 ± 0.56	21.09 ± 0.55	21.36 ± 0.56	20.73 ± 0.56	0.80
Thiamin, mg	2.29 ± 0.03	2.10 ± 0.03	1.94 ± 0.03	1.78 ± 0.03	< 0.001	2.12 ± 0.36	2.08 ± 0.35	1.96 ± 0.35	1.96 ± 0.36	0.01
Vitamin B6, mg	1.71 ± 0.04	1.79 ± 0.04	1.92 ± 0.04	1.77 ± 0.04	0.01	1.67 ± 0.04	1.73 ± 0.04	1.88 ± 0.04	1.91 ± 0.04	< 0.001
Iron, mg	20.38 ± 0.33	18.33 ± 0.31	17.06 ± 0.32	15.57 ± 0.32	< 0.001	18.62 ± 0.35	18.39 ± 0.34	17.57 ± 0.34	16.71 ± 0.34	< 0.001
Food groups										
Whole grains, g/d	136.75 ± 9.58	105.63 ± 7.23	77.28 ± 5.24	60.97 ± 3.87	< 0.001	126.11 ± 8.19	104.58 ± 7.92	87.91 ± 6.49	61.18 ± 4.58	< 0.001
Refined grains, g/d	331.61 ± 20.46	289.83 ± 14.82	225.64 ± 10.14	244.14 ± 12.30	< 0.001	306.64 ± 19.93	280.37 ± 14.06	251.74 ± 12.40	251.47 ± 13.11	0.02
Fruits, g/d	598.22 ± 40.27	588.71 ± 32.73	558.11 ± 30.57	471.18 ± 26.66	0.02	589.06 ± 35.99	538.79 ± 33.44	576.84 ± 28.93	510.86 ± 33.38	0.32
Vegetables, g/d	313.89 ± 21.21	335.73 ± 21.07	358.32 ± 24.50	336.74 ± 20.28	0.56	262.10 ± 17.95	335.50 ± 18.06	373.57 ± 24.02	374.52 ± 25.08	< 0.001
Red and processed meat, g/d	43.39 ± 2.57	61.76 ± 3.15	80.63 ± 4.75	85.29 ± 4.95	< 0.001	67.04 ± 5.28	60.49 ± 3.96	75.48 ± 3.95	68.45 ± 3.44	0.09
Dairy, g/d	215.95 ± 13.15	313.72 ± 22.60	352.41 ± 28.31	377.12 ± 28.20	< 0.001	291.93 ± 29.19	292.97 ± 21.32	349.21 ± 24.02	326.89 ± 22.34	0.27
Nuts and legumes, g/d	45.63 ± 3.311	51.25 ± 3.70	53.95 ± 3.51	52.73 ± 3.47	0.35	51.25 ± 3.78	54.98 ± 4.11	49.08 ± 3.05	48.25 ± 2.92	0.53

^a^Values are mean ± SE. Intakes of energy and macronutrients were adjusted for age and sex, all other values are adjusted for age, sex and energy intake.

^b^*P*-values obtained from ANCOVA.

SFA, Saturated fatty acids; MUFA, Monounsaturated fatty acids; PUFA, Polyunsaturated fatty acids.

**Figure 1:**
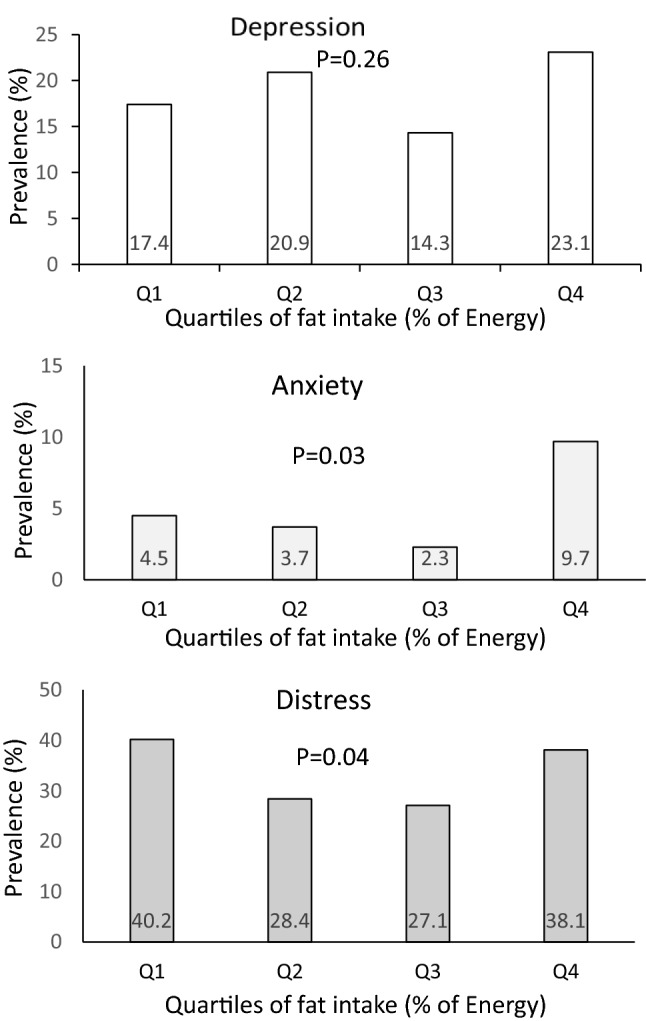
Prevalence of psychological disorders across quartiles of fat intake (as percentage of energy intake).

**Figure 2:**
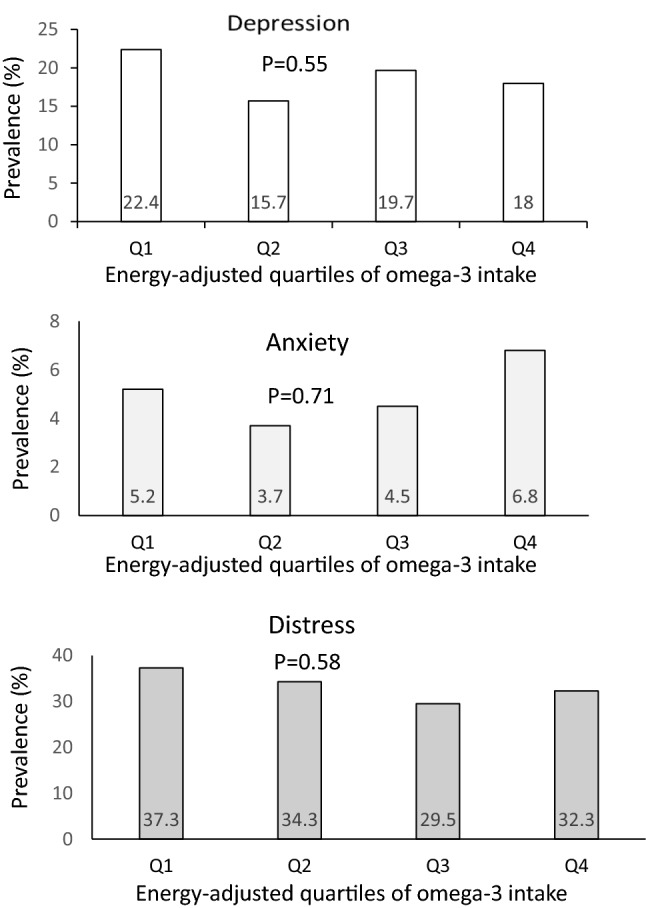
Prevalence of psychological disorders across energy-adjusted quartiles of omega-3 intake.

The prevalence of psychological disorders in study participants across quartiles of fat intake is shown in Fig. 1. A U-shaped relationship between fat intake and prevalence of anxiety was observed (*P* = 0.03); such that when fat intake was increased from the first quartile (18.12% of energy intake) to the third quartile (28.90% of energy intake), prevalence of anxiety was declined. However, prevalence of anxiety increased in the last quartile of fat intake (36.12% of energy intake). Similarly, a U-shaped relationship was found between fat intake and prevalence of distress (*P* = 0.04); such that when fat intake increased from the first category of fat intake to the third category, a decreasing trend in prevalence of distress was found; whereas, prevalence of distress increased in the last category of fat intake. Prevalence of psychological disorders among study participants across quartiles of energy-adjusted omega-3 fatty acids intake is provided in Fig. 2. No significant difference was found in prevalence of depression (*P* = 0.55), anxiety (*P* = 0.71) or distress (*P* = 0.58) across quartiles of omega-3 fatty acids intake.

Multivariate adjusted odds ratio (OR) and 95% confidence interval (CI) for psychological disorders among study participants across quartiles of fat and energy-adjusted omega-3 fatty acids intake are provided in Table 3. After adjustment for potential confounders, those in the third quartile of fat intake compared to the first one had 80% lower odds of depression (OR = 0.20, 95% CI 0.05–0.80). Furthermore, participants in the third quartile of fat intake in comparison to those in the first quartile had significantly 45% lower odds of distress, in the crude model (OR = 0.55, 95% CI 0.33–0.92). However, this association disappeared after adjustments for all potential confounders (OR = 0.61, 95% CI 0.25–1.48). Fat intake was not significantly related to anxiety, in crude or fully-adjusted models. In case of omega-3 fatty acids intake, there was no significant association between omega-3 fatty acids intake and odds of depression, anxiety and distress, in crude model. After adjustments for potential confounders, inverse associations between omega-3 fatty acids intake and psychological disorders were observed (for depression: OR _Q4 vs. Q1_ = 0.78, 95% CI 0.27–2.20; for anxiety: OR _Q4 vs. Q1_ = 0.45, 95% CI 0.04–4.3; for distress: OR _Q4 vs. Q1_ = 0.63, 95% CI 0.27–1.47); but these associations were not statistically significant.

**Table 3:** Multivariate adjusted odds ratio (OR) and 95% confidence interval (CI) for mental disorders in quartiles of fat (as percentage of energy intake) and energy-adjusted omega-3 fatty acids intake^a^.

	Quartiles of fat intake (% of energy)					Energy-adjusted quartiles of omega-3 fatty acids intake (g/d)				
	Q1 (n = 132) (< 21.8%)	Q2 (n = 134) (21.8–26.7%)	Q3 (n = 133) (26.7–31.4%)	Q4 (n = 134) (> 31.4%)	P trend	Q1 (n = 134) (< 0.08 g)	Q2 (n = 134) (0.08–0.13 g)	Q3 (n = 132) (0.13–0.19 g)	Q4 (n = 133) (> 0.19 g)	P trend
Mean fat or omega-3 fatty acids intake	18.12 ± 2.9	24.39 ± 1.4	28.90 ± 1.2	36.12 ± 4.0		0.06 ± 0.01	0.10 ± 0.01	0.15 ± 0.01	0.27 ± 0.10	
Depression										
Cases	23	28	19	31	0.26	30	21	26	24	0.55
Crude	1.00 (Ref)	1.25 (0.67, 2.31)	0.79 (0.40, 1.53)	1.42 (0.78, 2.60)	0.49	1.00 (Ref)	0.64 (0.34, 1.19)	0.85 (0.47, 1.53)	0.76 (0.41, 1.39)	0.55
Model 1	1.00 (Ref)	1.10 (0.58, 2.09)	0.62 (0.31, 1.25)	1.15 (0.60, 2.19)	0.97	1.00 (Ref)	0.57 (0.30, 1.09)	0.72 (0.38, 1.34)	0.59 (0.31, 1.12)	0.19
Model 2	1.00 (Ref)	1.42 (0.51, 3.89)	0.20 (0.05, 0.78)	0.95 (0.32, 2.80)	0.43	1.00 (Ref)	0.84 (0.30, 2.30)	0.69 (0.25, 1.89)	0.72 (0.26, 2.00)	0.49
Model 3	1.00 (Ref)	1.37 (0.49, 3.79)	0.20 (0.05, 0.80)	1.01 (0.33, 3.01)	0.52	1.00 (Ref)	0.81 (0.29, 2.24)	0.69 (0.25, 1.92)	0.78 (0.27, 2.20)	0.61
Anxiety										
Cases	6	5	3	13	0.03	7	5	6	9	0.71
Crude	1.00 (Ref)	0.81 (0.24, 2.73)	0.48 (0.11, 1.98)	2.25 (0.83, 6.12)	0.10	1.00 (Ref)	0.70 (0.21, 2.27)	0.86 (0.28, 2.64)	1.31 (0.47, 3.64)	0.52
Model 1	1.00 (Ref)	0.73 (0.21, 2.51)	0.42 (0.10, 1.77)	1.89 (0.65, 5.51)	0.19	1.00 (Ref)	0.64 (0.19, 2.11)	0.75 (0.24, 2.34)	1.10 (0.38, 3.16)	0.75
Model 2	1.00 (Ref)	0.25 (0.02, 3.18)	-^2^	1.75 (0.25,12.08)	0.45	1.00 (Ref)	-^3^	0.61 (0.09, 3.97)	0.42 (0.04, 4.00)	0.82
Model 3	1.00 (Ref)	0.25 (0.02, 3.19)	-^2^	1.88 (0.26,13.44)	0.42	1.00 (Ref)	-^3^	0.58 (0.09, 3.81)	0.45 (0.04, 4.38)	0.83
Distress										
Cases	53	38	36	51	0.04	50	46	39	43	0.58
Crude	1.00 (Ref)	0.59 (0.35. 0.98)	0.55 (0.33, 0.92)	0.91 (0.56, 1.49)	0.69	1.00 (Ref)	0.87 (0.53, 1.44)	0.70 (0.42, 1.17)	0.80 (0.48, 1.32)	0.28
Model 1	1.00 (Ref)	0.56 (0.33, 0.95)	0.50 (0.29, 0.86)	0.85 (0.50, 1.45)	0.55	1.00 (Ref)	0.84 (0.51, 1.41)	0.68 (0.40, 1.16)	0.74 (0.44, 1.25)	0.20
Model 2	1.00 (Ref)	0.77 (0.34, 1.75)	0.60 (0.25, 1.48)	0.93 (0.40, 2.17)	0.83	1.00 (Ref)	0.76 (0.33, 1.73)	0.59 (0.25, 1.35)	0.64 (0.27, 1.47)	0.25
Model 3	1.00 (Ref)	0.78 (0.34, 1.76)	0.61 (0.25, 1.48)	0.93 (0.40, 2.17)	0.83	1.00 (Ref)	0.76 (0.34, 1.74)	0.59 (0.25, 1.35)	0.63 (0.27, 1.47)	0.24

^a^All values are odds ratios and 95% confidence intervals. P-trend was obtained by the use of fat or omega-3 quartiles as a continuous rather than categorical variable. Model 1: Adjusted for age, sex, and energy. Model 2: Additionally, adjusted for marital status, education, physical activity, smoking, hypertension, diabetes, taking anti-depressive drugs, taking dietary supplements (including vitamin D, omega-3, B complex, vitamin B1, B2, B3, B6, multivitamin, neurobion, calcium and iron) and socioeconomic status (SES). Model 3: Additionally, adjusted for body mass index (BMI).

^b^OR (95% CI) could not be calculated, due to not having enough cases in this quartile.

**Figure 3:**
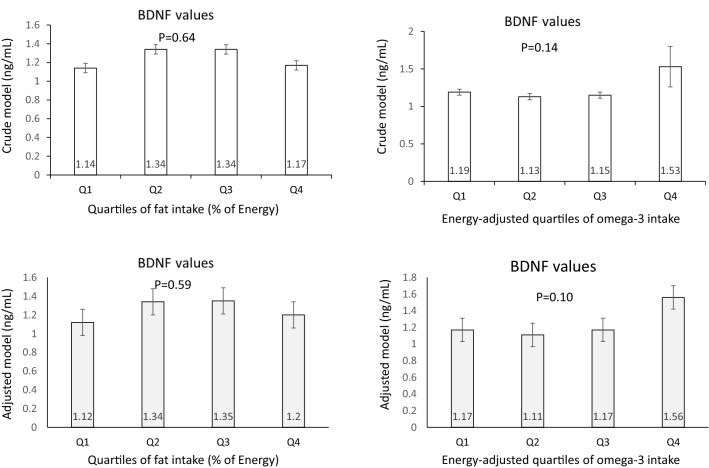
Crude and adjusted serum BDNF values (mean ± SE) across quartiles of fat (as percentage of energy intake) and energy-adjusted omega-3 intake.

Crude and adjusted serum BDNF values of study participants across quartiles of fat and omega-3 fatty acids intakes are provided in Fig. 3. Although individuals with moderate intake of fat (those in quartiles 2 and 3) had higher serum BDNF concentrations than subjects with the highest or the lowest fat intake (those in quartiles 4 and 1), there was no significant difference in crude values of serum BDNF across quartiles of fat intake (*P* = 0.64) and omega-3 fatty acids intake (*P* = 0.14). After adjustments for potential confounders, no significant difference in values of serum BDNF across quartiles of fat intake (*P* = 0.59) or omega-3 fatty acids intakes (*P* = 0.10) was found.

**Figure 4:**
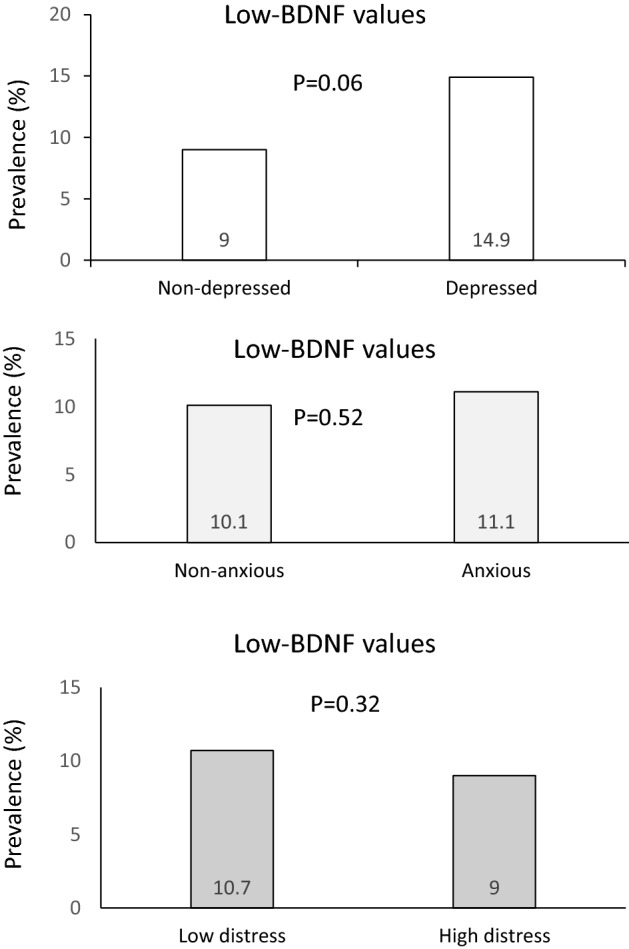
Prevalence of low-BDNF values in participants with psychological disorders compared to healthy subjects.

Prevalence of low-BDNF values in study participants with and without psychological disorders is presented in Fig. 4. Prevalence of low-BDNF values was higher in participants with depression, as compared to non-depressed subjects (14.9% vs. 9%; *P* = 0.06). Prevalence of low-BDNF values in participants with anxiety (*P* = 0.52) and psychological distress (*P* = 0.32) was not significantly different from healthy subjects.

## Discussion

In the current population-based cross-sectional study, there was a U-shaped relationship between fat intake and prevalence of anxiety and distress. Although increasing fat intake from low levels (Q1: 18.12 percentage of energy intake) to moderate intake (Q3: 28.90 percentage of energy intake) was related to a steeper reduction in prevalence of anxiety and distress, higher fat intake (Q4: 36.12% of percentage of energy intake) was associated with an increased prevalence of anxiety and distress. Furthermore, moderate fat intake in comparison to low intake (Q3 vs. Q1) was related to lower odds of depression. Additionally, participants who suffered from depression had slightly higher odds for low BDNF values, compared to non-depressed subjects. However, there was no significant relationship between omega-3 fatty acids intake and psychological disorders. No significant association was found between fat or omega-3 fatty acids intake and BDNF values.

The current investigation showed that a considerable percentage of Iranian adults suffered from depression (18.9%), anxiety (5.1%), and distress (33.4%). These mental disorders have adverse effects on quality of life and can increase risk of non-communicable diseases (NCDs)^35^. We found a U-shaped association between fat intake and prevalence of mental disorders. Therefore, it could be clinically recommended to Iranian people to keep their fat intake in the range of 26–31% of their energy intake to decrease risk of psychological disorders.

The relationship between macronutrient intake and risk of depression was investigated in several prior studies. In a recently published cross-sectional study, databases of National Health and Nutrition Examination Survey (NHANES) and South Korea NHANES (K-NHANES) were used for this assessment. Protein intake was inversely associated with odds of depression. Additionally, carbohydrate intake was related to increased odds of depression, only in the United States population. Nevertheless, there was no association between fat intake and odds of depression^36^. A similar cohort analysis also investigated the association between fatty acid intake and risk of depression in Spanish university graduates (SUN Project). This investigation illustrated a direct relationship between fat intake and risk of depression. However, an inverse weak association was found between MUFA, PUFA and olive oil and risk of depression^37^. As far as we know, our study was the first investigation that found a U-shaped relationship between fat intake and prevalence of anxiety and distress. In addition, moderate fat intake was associated with decreased odds of depression. Another analysis examined the relation of omega-3 fatty acids intake with anxiety disorder, by using Brazilian Longitudinal Study of Adult Health (ELSA-Brasil) database. The mentioned cross-sectional study illustrated an inverse association between EPA, DHA, docosapentaenoic acid (DPA) and anxiety disorders. Additionally, the n−6/n−3 ratio was straightly associated with anxiety disorder; however, these relationships disappeared after adjustments for multiple comparisons^25^. Similarly, we could not find any significant relationship between omega-3 fatty acids intake and psychological disorders in crude and adjusted models.

A randomized clinical trial demonstrated that 1 g/d ethyl-eicosapentaenoic acid (E-EPA) could not significantly improve serum BDNF levels in patients with diabetes and major depression^38^. However, an 8-week omega-3 fatty acids supplementation in male patients with coronary artery disease (CAD) had a favorable effect on BDNF values, serum high-sensitivity C-reactive protein (hs-CRP) and low-density lipoprotein cholesterol (LDL-c)^39^. Furthermore, omega-3 fatty acids supplementation elevated BDNF levels in diabetic patients, while no significant effect on anthropometric indices was found^29^. Although several RCTs have examined the effect of omega-3 fatty acids supplementation on circulating BDNF concentrations, the relation of usual dietary fat intake or omega-3 fatty acids intake with serum BDNF values has been understudied so far. The current epidemiologic study has investigated the relation of usual long-standing dietary fat and omega-3 fatty acid intakes with serum BDNF values and we found that moderate intake of fat might be associated with higher serum BDNF levels. Further studies with prospective design are needed to shed a light on this association.

Several mechanisms have been proposed to clarify the favorable or unfavorable effects of fat intake on psychological disorders. Fatty acids are important components of CNS in the human body. The fat content of cell membrane can influence mood status^15,16^. Moreover, oleic acid content of diet is a biologically active fatty acid that can be related to a lower risk of depression^40^. Furthermore, omega-3 PUFAs are involved in signal transmission, receptor function, neurotransmitter reuptake and could increase serotonin transportation by increasing the membrane fluidity ^41^. Inflammatory factors play an important role in the synthesis, processing, and transmission of neurotransmitters (such as serotonin, glutamate, and dopamine)^42^. Prior evidences illustrated that mental disorders such as depression are associated with an inflammatory response of the immune system and increased inflammatory cytokines^43^. However, some fatty acids, especially omega-3 fatty acids could inhibit expression of cytokines such as tumor necrosis factor-alpha (TNF-α) and interleukin-1beta (IL-1β) ^44^.

On the other hand, the current analysis documented that high fat intake as compared to moderate intake was related to an increased risk of psychological disorders. It is worth noting that lipid peroxidation and inflammatory response -due to high intake of fat- are drastically involved in incidence of mental disorders^45^. Additionally, an unhealthy diet with high-fat content is a poor source of vitamins, minerals, polyphenols and fibers, which are involved in neurogenesis and neuroprotection^12^. High-fat foods such as processed foods are rich in saturated fatty acids which can increase symptoms of mental disorders through the gut microbiota and inflammatory pathways^12^. Additionally, it should be considered that inflammation and mental disorders due to a high-fat diet might have adverse effect on serum BDNF concentrations, as a mediator molecule^46^.

The current study has some strengths and weaknesses. This investigation was conducted on a representative sample of adults with various socioeconomic statuses; therefore, the findings could be extrapolated to the whole adult population. Psychological disorders and dietary intakes were examined by the use of validated questionnaires. Moreover, the effects of several potential confounders were controlled in the analyses. Nevertheless, there were some limitations that should be considered while interpreting the findings. Due to cross-sectional design of the study, we could not deduce a causal relationship between fat or omega-3 fatty acids intake and psychological disorders. More prospective studies should be performed to affirm causality. Although a validated FFQ was used to assess dietary intake, recall bias might influence the findings. Applying a self-reporting FFQ might also lead to misclassification of individuals among categories of fat or omega-3 fatty acids intake. In addition, the current study was conducted in a central province of Iran (Isfahan), where consumption of omega-3-rich sources such as sea foods was very low. Thus, the range of omega-3 fatty acids intake among quartiles was too narrow (first vs. last quartile: 0.06 vs. 0.27 g/d); this point might result in null observed associations between omega-3 intake and outcomes of interest. Although this study was conducted on a general population, selecting participants from an organization could affect the generalizability of the findings. Finally, the effect of residual confounders should also be taken into account.

In conclusion, this cross-sectional study illustrated a U-shaped relationship between fat intake and prevalence of anxiety and distress. Additionally, moderate intake of fat was related to lower odds of depression. Furthermore, prevalence of low-BDNF values was slightly higher in subjects with depression compared to non-depressed individuals. Further studies with prospective design are required to confirm these findings.

## Data Availability

The data that support the findings of this study are available from the corresponding author upon reasonable request.

## Abbreviations

FFQ: Food frequency questionnaire
OR: Odds ratios
95% CI: 95% confidence interval
BMI: Body mass index
MUFA: Mono unsaturated fatty acid
PUFA: Poly unsaturated fatty acid
SFA: Saturated fatty acid
ANOVA: Analysis of variance
ANCOVA: Analysis of covariance
SPSS: Statistical package for the social sciences
SD: Standard deviation
SE: Standard error
NCD: Non-communicable diseases
BDNF: Brain-derived neurotrophic factor
CNS: Central nervous system
NGF: Nerve growth factor
RCT: Randomized clinical trial
GHQ: General health questionnaire
HADS: Hospital anxiety and depression scale
DHA: Docosahexaenoic acid
EPA: Eicosapentaenoic acid
ALA: Alpha-linolenic acid
DPA: Docosapentaenoic acid
E-EPA: Ethyl-eicosapentaenoic acid
CAD: Coronary artery disease
LDL-c: Low-density lipoprotein cholesterol
IL-1b: Interleukin-1 beta
TNF-a: Tumor necrosis factor-alpha
SES: Socioeconomic status
IPAQ: International physical activity questionnaire
NHANES: National health and nutrition examination survey
K-NHANES: South Korea NHANES
hs-CRP: High-sensitivity C-reactive protein
CVD: Cardiovascular disease
MI: Myocardial infarction

## References

[1] Baxter AJ, Scott KM, Vos T, Whiteford HA (2013). Global prevalence of anxiety disorders: a systematic review and meta-regression. Psychol. Med..

[2] Ferrari AJ, Somerville AJ, Baxter AJ, Norman R, Patten SB, Vos T, Whiteford H (2013). Global variation in the prevalence and incidence of major depressive disorder: a systematic review of the epidemiological literature. Psychol. Med..

[3] Steel Z, Marnane C, Iranpour C, Chey T, Jackson JW, Patel V (2014). The global prevalence of common mental disorders: a systematic review and meta-analysis 1980–2013. Int. J. Epidemiol..

[4] Saneei P, Esmaillzadeh A, Keshteli AH, Roohafza HR, Afshar H, Feizi A, Adibi P (2021). Combined healthy lifestyle is inversely associated with upper gastrointestinal disorders among Iranian adults. Digest. Dis..

[5] Bauer ME, Teixeira AL (2019). Inflammation in psychiatric disorders: what comes first?: Inflammation in psychiatric disorders. Ann. N. Y. Acad. Sci..

[6] Nemeroff CB, Goldschmidt-Clermont PJ (2012). Heartache and heartbreak—the link between depression and cardiovascular disease. Nat. Rev. Cardiol..

[7] Kanwar A, Malik S, Prokop LJ, Sim LA, Feldstein D, Wang Z, Murad MH (2013). The association between anxiety disorders and suicidal behaviors: a systematic review and meta-analysis. Depress. Anxiety.

[8] Kanwar A, Malik S, Prokop LJ, Sim LA, Feldstein D, Wang Z, Murad MH (2013). The association between anxiety disorders and suicidal behaviors: a systematic review and meta-analysis. Depress. Anxiety.

[9] Gilman SE, Sucha E, Kingsbury M, Horton NJ, Murphy JM, Colman I (2017). Depression and mortality in a longitudinal study: 1952–2011. Can. Med. Assoc. J..

[10] Mullins N, Lewis CM (2017). Genetics of depression: progress at last. Curr. Psychiatry Rep..

[11] Gold PW (2021). Endocrine factors in key structural and intracellular changes in depression. Trends Endocrinol. Metab..

[12] Firth J, Marx W, Dash S, Carney R, Teasdale SB, Solmi M (2019). The effects of dietary improvement on symptoms of depression and anxiety: a meta-analysis of randomized controlled trials. Psychosomat. Med..

[13] Null G, Pennesi L (2017). Diet and lifestyle intervention on chronic moderate to severe depression and anxiety and other chronic conditions. Compl. Therap. Clin. Pract..

[14] Jacka FN, Mykletun A, Berk M, Bjelland I, Tell GS (2011). The association between habitual diet quality and the common mental disorders in community-dwelling adults: the Hordaland Health study. Psychosomat. Med..

[15] Müller CP, Reichel M, Mühle C, Rhein C, Gulbins E, Kornhuber J (2015). Brain membrane lipids in major depression and anxiety disorders. Biochim. Biophys. Acta (BBA) Mol. Cell Biol. Lipids.

[16] Larrieu T, Layé S (2018). Food for mood: Relevance of nutritional omega-3 fatty acids for depression and anxiety. Front. Physiol..

[17] Lange, K. W. & Nakamura, Y. The role of omega-3 polyunsaturated fatty acids in mental disorders. **4** (2020).

[18] Godos, J., Castellano, S., Galvano, F. & Grosso, G. Linking omega-3 fatty acids and depression. In *Omega Fatty Acids in Brain and Neurological Health* 199–212. (Academic Press, 2019).

[19] McNamara RK (2016). Role of omega-3 fatty acids in the etiology, treatment, and prevention of depression: current status and future directions. J. Nutr. Intermed. Metabol..

[20] Park H, Poo MM (2013). Neurotrophin regulation of neural circuit development and function. Nat. Rev. Neurosci..

[21] Suliman S, Hemmings SM, Seedat S (2013). Brain-Derived Neurotrophic Factor (BDNF) protein levels in anxiety disorders: systematic review and meta-regression analysis. Front. Integ. Neurosci..

[22] Bus BAA, Molendijk ML, Tendolkar I, Penninx BWJH, Prickaerts J, Elzinga BM, Voshaar RO (2015). Chronic depression is associated with a pronounced decrease in serum brain-derived neurotrophic factor over time. Mol. Psychiatry.

[23] Björkholm C, Monteggia LM (2016). BDNF–a key transducer of antidepressant effects. Neuropharmacology.

[24] Grosso G, Micek A, Marventano S, Castellano S, Mistretta A, Pajak A, Galvano F (2016). Dietary n-3 PUFA, fish consumption and depression: a systematic review and meta-analysis of observational studies. J. Affect. Disord..

[25] Natacci L, Marchioni M (2018). Omega 3 consumption and anxiety disorders: a cross-sectional analysis of the brazilian longitudinal study of adult health (ELSA-Brasil). Nutrients.

[26] Hakkarainen R, Partonen T, Haukka J, Virtamo J, Albanes D, Lönnqvist J (2004). Is low dietary intake of omega-3 fatty acids associated with depression?. Am. J. Psychiatry.

[27] Timonen M, Horrobin D, Jokelainen J, Laitinen J, Herva A, Räsänen P (2004). Fish consumption and depression: the Northern Finland 1966 birth cohort study. J. Affect. Disord..

[28] Hadjighassem M, Kamalidehghan B, Shekarriz N, Baseerat A, Molavi N, Mehrpour M (2015). Oral consumption of α-linolenic acid increases serum BDNF levels in healthy adult humans. Nutr. J..

[29] Ansari S, Djalali M, Mohammadzadeh N, Mohammadzadeh Honarvar N, Mazaherioun M, Zarei M (2016). Assessing the effect of omega-3 fatty acids supplementation on serum BDNF (brain derived neurotrophic factor) in patients with type 2 diabetes: a randomized, double-blind, placebo-controlled study. Int. Res. J. Appl. Basic Sci..

[30] Mirmiran P, Esfahani FH, Mehrabi Y, Hedayati M, Azizi F (2010). Reliability and relative validity of an FFQ for nutrients in the Tehran lipid and glucose study. Public Health Nutr..

[31] Montazeri A, Vahdaninia M, Ebrahimi M, Jarvandi S (2003). The Hospital Anxiety and Depression Scale (HADS): translation and validation study of the Iranian version. Health Quality Life Outcomes.

[32] Montazeri A, Harirchi AM, Shariati M, Garmaroudi G, Ebadi M, Fateh A (2003). The 12-item General Health Questionnaire (GHQ-12): translation and validation study of the Iranian version. Health Quality Life Outcomes.

[33] Sánchez-Villegas A, Galbete C, Martinez-González MÁ, Martinez JA, Razquin C, Salas-Salvadó J (2011). The effect of the Mediterranean diet on plasma brain-derived neurotrophic factor (BDNF) levels: the PREDIMED-NAVARRA randomized trial. Nutr. Neurosci..

[34] Moghaddam MB, Aghdam FB, Jafarabadi MA, Allahverdipour H, Nikookheslat SD, Safarpour S (2012). The Iranian Version of International Physical Activity Questionnaire (IPAQ) in Iran: content and construct validity, factor structure, internal consistency and stability. World Appl. Sci. J..

[35] Stein, D. J., Benjet, C., Gureje, O., Lund, C., Scott, K. M., Poznyak, V. & Van Ommeren, M. Integrating mental health with other non-communicable diseases. *Bmj*, **364**. (2019). 10.1136/bmj.l295 PMC6348425 30692081

[36] Oh J, Yun K, Chae JH, Kim TS (2020). Association between macronutrients intake and depression in the United States and South Korea. Front. Psychiatry.

[37] Sánchez-Villegas A, Verberne L, De Irala J, Ruiz-Canela M, Toledo E, Serra-Majem L, Martínez-González MA (2011). Dietary fat intake and the risk of depression: the SUN Project. PloS One.

[38] Bot M, Pouwer F, Assies J, Jansen EH, Beekman AT, De Jonge P (2011). Supplementation with eicosapentaenoic omega-3 fatty acid does not influence serum brain-derived neurotrophic factor in diabetes mellitus patients with major depression: a randomized controlled pilot study. Neuropsychobiology.

[39] Agh, F., Honarvar, N. M., Djalali, M., Nematipour, E., Zarei, M. & Salim, S. J. et al. Brain-derived neurotrophic factor (BDNF) is increased by omega-3 fatty acids in coronary artery disease: a randomized, double-blind, placebo-controlled. 2–8. (2016).

[40] Li D, Tong Y, Li Y (2020). Associations between dietary oleic acid and linoleic acid and depressive symptoms in perimenopausal women: the Study of Women's Health Across the Nation. Nutrition.

[41] Fernstrom JD (1999). Effects of dietary polyunsaturated fatty acids on neuronal function. Lipids.

[42] Capuron L, Miller AH (2011). Immune system to brain signaling: neuropsychopharmacological implications. Pharmacol. Therapeut..

[43] Beurel E, Toups M, Nemeroff CB (2020). The bidirectional relationship of depression and inflammation: double trouble. Neuron.

[44] Calder PC (2017). Omega-3 fatty acids and inflammatory processes: from molecules to man. Biochem. Soc. Trans..

[45] Tsaluchidu S, Cocchi M, Tonello L, Puri BK (2008). Fatty acids and oxidative stress in psychiatric disorders. BMC Psychiatry.

[46] Motamedi S, Karimi I, Jafari F (2017). The interrelationship of metabolic syndrome and neurodegenerative diseases with focus on brain-derived neurotrophic factor (BDNF): kill two birds with one stone. Metab. Brain Dis..

